# Using Molecular Networking for Microbial Secondary Metabolite Bioprospecting

**DOI:** 10.3390/metabo6010002

**Published:** 2016-01-08

**Authors:** Kevin Purves, Lynsey Macintyre, Debra Brennan, Guðmundur Ó. Hreggviðsson, Eva Kuttner, Margrét E. Ásgeirsdóttir, Louise C. Young, David H. Green, Ruangelie Edrada-Ebel, Katherine R. Duncan

**Affiliations:** 1 Scottish Association for Marine Science, Scottish Marine Institute, Oban PA37 1QA, UK; kep@pml.ac.uk (K.P); debra.brennan@sams.ac.uk (D.B); david.green@sams.ac.uk (D.H.G.); 2 Strathclyde Institute of Pharmacy and Biomedical Sciences, University of Strathclyde, Glasgow G1 1XQ, UK; lynsey.macintyre@strath.ac.uk (L.M.); louise.young@strath.ac.uk (L.C.Y.); ruangelie.edra-ebel@strath.ac.uk (R.E.-E.); 3 Matis, Vinlandsleið 12, Reykjavik 113, Iceland; gudmundo@matis.is (G.O.H.); eva.kuttner@matis.is (E.K.); margreteva@matis.is (M.E.A); 4 Faculty of Life and Environmental Sciences, University of Iceland, 101 Reykjavik, Iceland

**Keywords:** molecular networking, secondary metabolites, bioprospecting, bacteria, Antarctica

## Abstract

The oceans represent an understudied resource for the isolation of bacteria with the potential to produce novel secondary metabolites. In particular, actinomyces are well known to produce chemically diverse metabolites with a wide range of biological activities. This study characterised spore-forming bacteria from both Scottish and Antarctic sediments to assess the influence of isolation location on secondary metabolite production. Due to the selective isolation method used, all 85 isolates belonged to the phyla Firmicutes and Actinobacteria, with the majority of isolates belonging to the genera *Bacillus* and *Streptomyces*. Based on morphology, thirty-eight isolates were chosen for chemical investigation. Molecular networking based on chemical profiles (HR-MS/MS) of fermentation extracts was used to compare complex metabolite extracts. The results revealed 40% and 42% of parent ions were produced by Antarctic and Scottish isolated bacteria, respectively, and only 8% of networked metabolites were shared between these locations, implying a high degree of biogeographic influence upon secondary metabolite production. The resulting molecular network contained over 3500 parent ions with a mass range of *m/z* 149–2558 illustrating the wealth of metabolites produced. Furthermore, seven fermentation extracts showed bioactivity against epithelial colon adenocarcinoma cells, demonstrating the potential for the discovery of novel bioactive compounds from these understudied locations.

## 1. Introduction

Secondary metabolites have had a profound impact on human health, nutrition and society, since their initial discovery in the 1920s [1]. These natural products have been utilized for a number of applications such as antiparasitic [2], antimalarial [3,4], immunosuppressant [5,6], anticancer [7,8,9] and antibiotic [10,11] applications. Recently, the rise of antimicrobial-resistant bacteria has provided motivation for novel bioactive compound discovery, as it has been recognised by the World Health Organization as a threat to human health [12,13].

Through sequencing, unprecedented levels of marine microbial diversity have been revealed [14] across a range of habitats, including Arctic soils [14,15], permafrost soil in Greenland [16], the Canadian high Arctic [17], marine invertebrates [18,19,20,21] and the deep sea [14,15]. Bacterial bioprospecting within these understudied marine environments has yielded novel bioactive compounds [22,23,24], including abysomycin C, a polycyclic polyketide isolated from a marine *Verrucosispora* strain with activity against methicillin resistant *Staphlycoccus aureus* [25] and salinosporamide A, a proteasome inhibitor isolated from *Salinispora tropica* [26]. Of the total microbial bioactive metabolites identified 45% are produced by Actinomycetales, and within this, a single genus (*Streptomyces*) is responsible for 75% of these metabolites [27]. Members of the spore-forming genus *Bacillus* have also demonstrated versatile secondary metabolite production with diverse structural features and biological activities [28,29,30]. The lack of natural product discovery from Antarctica is likely as a result of the challenges associated with sampling from this region [31]. However, due to the ability of *Bacilli* and many actinomycetes to reside as spores, obtaining sediment samples from Antarctic depths could yield actinomycetes, as was found by the Challenger Deep Expedition in 2006, which cultivated thirty-eight actinomycetes from the Mariana Trench (10898 m) [32]. As a result, the isolation of novel bacteria from underexplored localities (*i.e.*, Scotland and Antarctica) is a potential approach for uncovering novel bioactive compounds.

Bacteria have the ability to adapt to extreme parameters, including, temperature, salinity and pH, which can result in ecologically-adapted populations residing in niche geographic habitats [14,32,33,34,35,36]. Horizontal gene transfer has been shown to be an important driver of bacterial diversification, whereby genes involved in natural product biosynthesis are swapped between taxa, resulting in great chemical diversity [37,38]. Over time, this adaptation can result in distinct phylogenetic taxa and the evolution of secondary metabolite biosynthetic genes in response to these ecosystem parameters. Therefore, understudied or extreme environments have the potential capacity to be a source of novel natural products [25,39,40].

Molecular networking allows rapid comparison of mass spectrometry profiles from complex crude fermentation extracts, for effective chemical dereplication and novel metabolite discovery. Molecular networks utilize high-resolution mass spectrometry parent ion fragmentation data (MS/MS) [41]. These parent ions are connected based on the chemical architecture of the compound. Similar compounds have similar parent ion fragmentation patterns, which is computed as a cosine score from 1 (identical fragmentation spectra) to 0 (completely different parent ions) [41,42]. The parent ions (nodes) are therefore connected by edges (cosine score), which results in analogous or structurally similar compounds grouping together in molecular families [41,43]. This is an exceptionally efficient and sensitive method for comparison of metabolite profiles and complex high-resolution mass spectrometry data. Molecular networking has been demonstrated for rapid chemical dereplication of complex crude fermentation extracts [44] and has been combined with pattern-based genome mining to assess the pan-metabolome of 35 *Salinispora* strains with the discovery of the novel compound retemycin A [45] and used in conjunction with glycogenomics [46] and peptidogenomics [47]. The comparative approach of chemical profiles has allowed changes in secondary metabolites in response to intraspecific interactions to be assessed for the cyanobacteria *Microcystis* [48] and it has been used to profile marine cyanobacteria metabolomes [49]. Molecular networking has also been combined with two and three-dimensional visualization of metabolites using real-time and imaging mass spectrometry [50] and stable isotope labelling for biosynthetic pathway characterization in fungi [51].

The current study establishes a taxonomically selective culture collection to investigate the potential of bacteria from both Scottish and Antarctic sediments to produce bioactive secondary metabolites. Culture-dependent approaches, utilising a variety of media and pre-treatments were employed to select for spore-forming bacteria (actinomyces and *Bacillus*), followed by taxonomic identification and phylogenetic analysis. Their secondary metabolites were investigated using tandem high-resolution mass spectrometry of fermentation extracts, followed by metabolite dereplication using molecular networking and bioactivity testing against epithelial colon adenocarcinoma cells.

## 2. Results and Discussion

### 2.1. Phylogenetic and Taxonomic Identification of Antarctic and Scottish Bacterial Isolates

Scottish sediments, sampled from three locations on the East coast and the Outer and Inner Hebrides, were used to isolate sixty-seven bacterial strains (Figure S1) following a selective cultivation approach encompassing five different media and two pre-treatments (Dilute Heat, DH; Dry Stamp, DS). The St. Andrews sediment yielded the highest number of isolates with 70.2% of all the Scottish bacteria isolated from this location using the DS pretreatment, followed by 7.5% using the DH pretreatment. The sediment taken from Calgary Bay yielded the second-highest number of isolates (13 bacteria, 19.4%). The isolation media generating the highest number of isolates was Starch Casein Nitrate (SC) (38.8% of the total isolates) followed by Raffinose-Histidine (RH) (17.9%). The combination of SC media, DS pre-treatment and St. Andrews sediment provided the highest number of bacterial isolates (26.9*%*). This supports previous studies that showed the highest number of actinomycetes were isolated from Canadian sediment using RH and SC media [23,52].

Antarctic sediments from four locations around the outer rim of the Weddell Sea were plated on five selective isolation media and yielded eighteen bacterial strains (Figure S2). The highest number of isolates was obtained from the Bransfield Strait (50.0*%*) of which seven strains (38.9*%*) were isolated using RH media. Wegener Canyon sediment that was plated on AI media and Bransfield Straight sediment that was plated on SC media yielded the highest number of isolates (22.2*%* each). Chitin Low Nutrient (M4) and Phosphate-Nitrate (M3) media yielded no bacterial isolates from any of the Antarctic samples. This is in contrast with two studies that cultured actinomycetes from marine sediments of Newfoundland and New Brunswick (Canada), as these media were shown to yield multiple bacterial isolates (M3: 59 and 113 isolates; M4: 41 and 19 isolates, respectively) [23,53]. Murray and Grzymski [54] found Actinobacteria were one of the main bacterial phylogenetic groups isolated from marine samples from Antarctica, which is likely due to the resilience of spore-forming bacteria to desiccation and sub-zero temperatures over extended time periods [55,56,57]. In this study, the prolonged time since the Antarctic (sampled 2002 and 2005) and Scottish (sampled 1990) sediments were collected may have impacted the number of bacteria isolated. To circumvent this issue, this study also investigated more recently sampled Scottish sediment (St. Andrews sediments, sampled in 2013) [58]. The selective isolation methods employed have been shown to yield high actinomycete diversity [58,59,60,61]. The lower number of Antarctic isolates may be a result of applying methods less amenable to bacterial isolation from extreme habitats that encompass abyssal depths, pressures and temperatures. However, Weyland *et al.* investigated actinomycete abundance of marine sediments from various locations and were unable to isolate any actinomycetes from Antarctic sediment [62], indicating that the methodology used herein may have contributed to the successful isolation.

**Figure 1:**
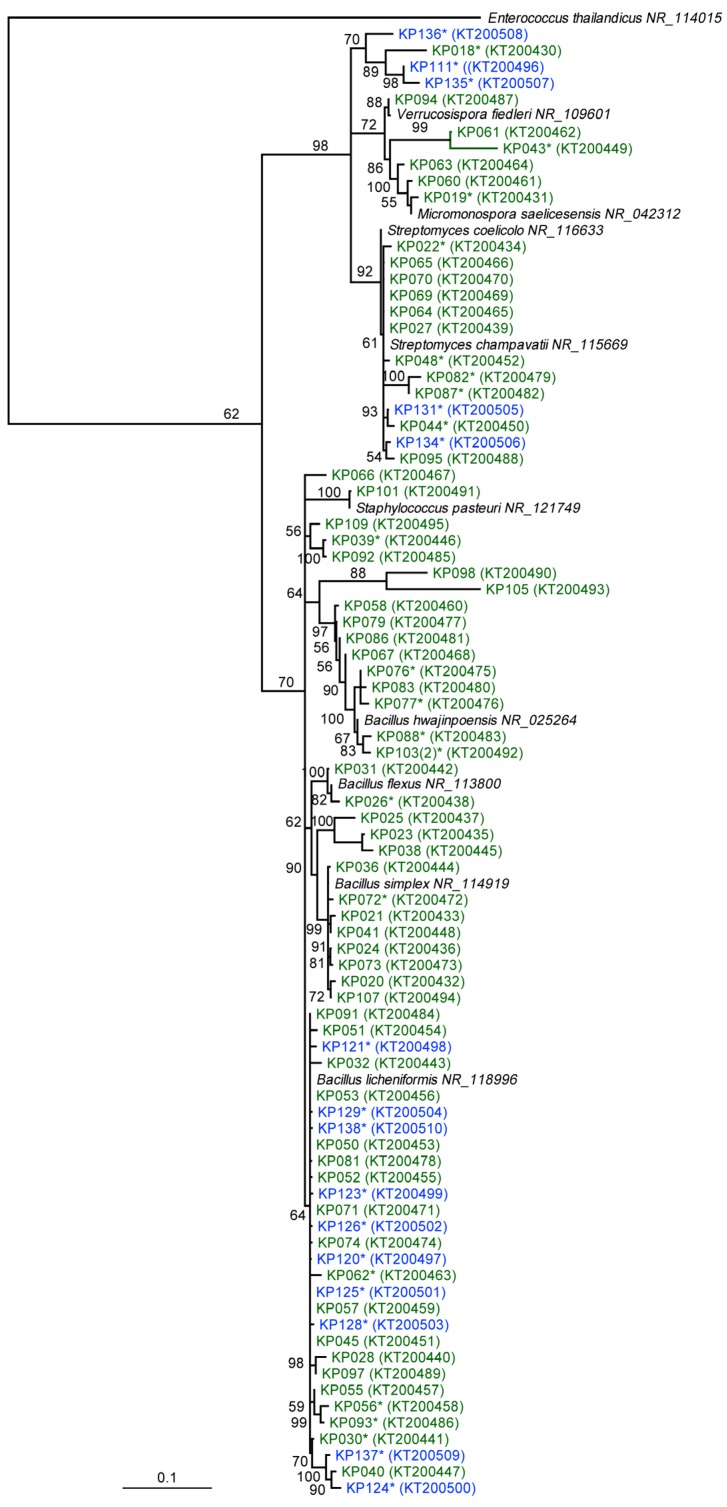
Neighbour-joining tree based on almost complete 16S rRNA gene sequences, showing the phylogenetic relationships between 81 Scottish and Antarctic bacterial isolates (KP011, KP122, KP130 and KP133 were excluded from the tree) with NCBI accession numbers in brackets. Bootstrap support >50*%* based on a neighbour-joining analysis of 1000 resampled datasets is shown. Scale bar, 0.1 substitutions per nucleotide position. * strains subjected to metabolite comparison through molecular networking. Branches are colour-coded according to their isolation location (Scotland, Green; Antarctica, Blue) for each bacterial isolate.

Furthermore, 16S rRNA gene sequencing of the 85 isolates revealed fine scale phylogenetic diversity (Figure 1) encompassing eight genera, including *Bacillus* (62.4*%*), *Streptomyces* (15.5*%*), *Micromonospora* (4.7*%*), *Paenibacillus* (4.7*%*), *Kocuria* (3.5*%*), *Verrucosispor*a (2.4*%*), *Staphylococcus* (1.2*%*), *Micrococcus* (1.2*%*) and four unidentified bacteria (4.7*%*) (Table S1). A total of thirty-eight isolates were chosen for chemical investigation based on morphology (Figure 1). These isolates were subsequently sequenced and belonged to the genera *Bacilllus* (11 Scottish, 10 Antarctic isolates), *Streptomyces* (5 Scottish, 2 Antarctic), *Kocuria* (3 Antarctic), *Micromonosprora* (2 Scottish), *Micrococcus* (1 Scottish) and four unidentified isolates from Antarctica (Table S1).

As a result of the targeted methodology to isolate spore-forming bacteria (*Bacillus* and actinomycetes) from Antarctic and Scottish sediment [27,58], the dominant genera cultured were *Bacillus* (62*%*) and *Streptomyces* (15*%*). A study of deep sea, anoxic brine lake sediments in the Eastern Mediterranean revealed *Bacillus*-like organisms to be the dominant cultured bacteria [63]. Similar to this study, a phylogenetic identification of marine bacteria isolated from deep-sea sediments from the South Atlantic Ocean yielded *Bacillus* as the most frequently identified genus [64]. In this study, strains from the rare actinomycete genus *Kocuria* were only isolated from the Antarctic sediments. Previously, *Kocuria* spp., isolated from Florida (USA), have been shown to produce antibacterial compounds (kocurin) effective against methicillin resistant *S. aureus* [65].

### 2.2. Comparative Metabolomics

**Figure 2:**
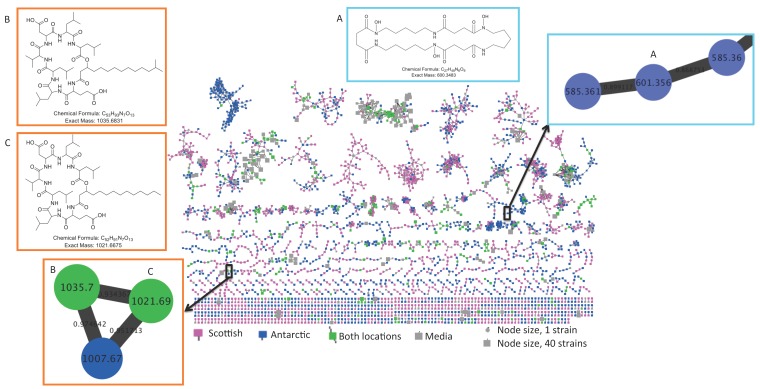
Molecular network of 3558 parent ions produced by 38 bacterial strains. Nodes colours are based on bacterial isolation location, where nodes representing parent ions are produced by strains isolated from Antarctic sediment, Scottish sediment or both. Grey nodes represent media components and the node size reflects the number of strains that produced each parent ion. Nodes highlighted in coloured boxes represent parent ions that are identified as the previously discovered metabolites (**A**) desferrioxamine E (**B**) surfactin C14 and (**C**) surfactin C15.

**Figure 3:**
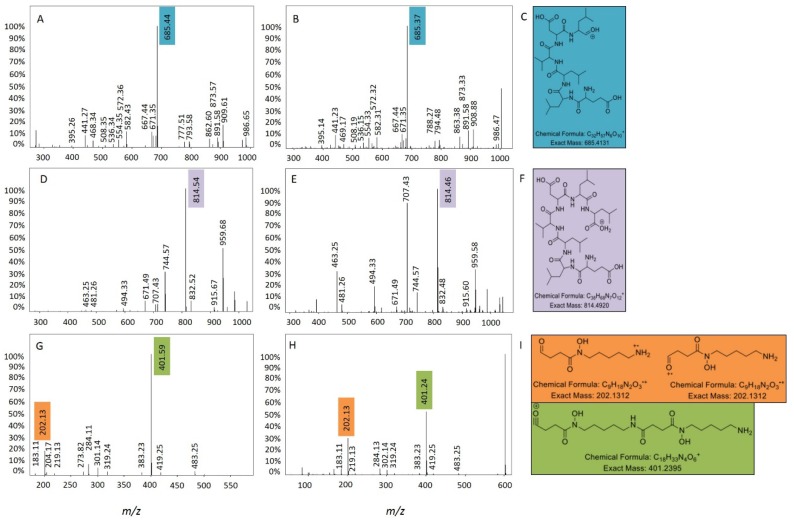
Surfactin C14 (**A**) MS/MS parent ion fragmentation for this study (**B**) MS/MS parent ion fragmentation for the molecular networking standard (**C**) major fragment ion; surfactin C15; (**D**) MS/MS parent ion fragmentation for this study; (**E**) MS/MS parent ion fragmentation for the molecular networking standard; (**F**) major fragment ion and desferrioxamine E (**G**) MS/MS parent ion fragmentation for this study; (**H**) MS/MS parent ion fragmentation for the molecular networking standard; (I) major fragment ions.

Molecular networking of metabolite mass spectrometry profiles from 38 bacteria was visualized by bacterial isolation location (Scotland and Antarctica) and resulted in a molecular network containing 3558 parent ions (nodes) (Figure 2). The node size indicates the number of strains producing the parent ion and as expected, media components have larger node sizes due to their ubiquitous presence in fermentation extracts (Figure 2). Parent ions within the molecular network only matched three known standards (within the molecular networking databases), the siderophore desferrioxamine E (*m/z* 601.358) produced by one Antarctic *Streptomyces* strain (KP131), surfactin C14 *(m/z* 1022.73*)* and surfactin C15 (*m/z* 1036.74) (both shown as non-charged ions in the molecular network) produced by four *Bacillus* strains, three from Scotland (KP056, KP088 and KP129) and one from Antarctica (KP129) (data not shown). For all three known compounds, example MS/MS parent ion fragmentation data from this study and the molecular networking standards were compared (Figure 3).

**Figure 4:**
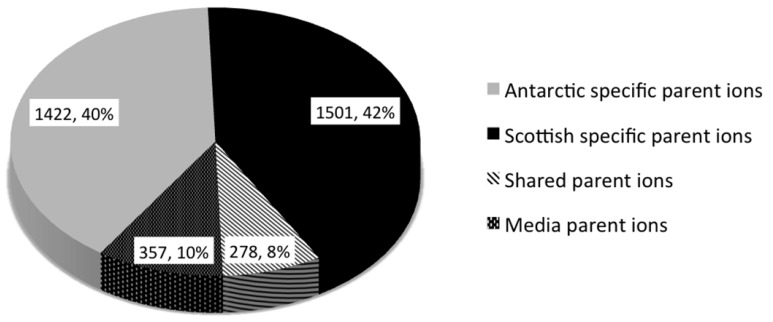
Relative proportions of location-specific parent ions identified by molecular network analysis (Figure 2).

The number of parent ions produced (*i.e.*, not media components) varied from strain specific production (79.4*%* parent ions in the molecular network) to production of parent ions by 10 strains. No parent ion was produced by all strains. This strain specificity yields great potential for the discovery of bioactive metabolites, whereby each strain contributes a high percentage of unique chemistry. The molecular network revealed that 2923 (82*%*) of parent ions were unique to either the Antarctic or Scottish isolates. A total of 1422 metabolites (39.9*%* of the total ions in the molecular network) were Antarctic specific secondary metabolites, whilst 1501 (42.1*%*) of metabolites were Scottish-specific. Remarkably, only 278 ions (7.8*%*) were produced by isolates from both locations (Figure 4). This contradicts previous observations of species specific metabolite production from the genus *Salinispora* [66].

**Figure 5:**
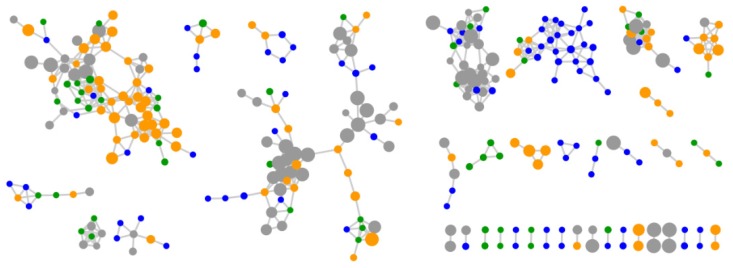
Molecular network with 315 parent ions produced by ten *Bacullus licheniformis* strains. Nodes representing ions produced by strains isolated from Scotland (KP30, KP30, KP56, KP62, KP93) and Antarctica (KP120, KP121, KP126, KP126, KP128) are green and blue, respectively. Nodes representing parent ions produced by strains isolated from both Scotland and Antarctica are orange and media components are grey. The node size reflects the number of strains that produce each parent ion from the smallest node (strain specific) to the largest node (10 strains).

To address this further, ten *Bacillus lichenformis* strains (five isolated from both Scotland and Antarctica) sharing >98*%* 16S rRNA gene sequence identities were selected for molecular networking, resulting in 315 parent ions (Figure 5). In total 43.8*%* of the *B. lichenformis* produced parent ions were specific to either Scotland (16.2*%*) or Antarctica (27.6*%*), which is a lower number than when all taxa were compared. In addition, the number of ions shared between these locations was 29.5*%*, again, higher than the 8*%* shared when comparing all bacterial taxa. In order to compare additional species, a higher number of highly related species or strains (>98*%* 16S rRNA gene sequence similarity) would be required. It is, however, surprising that although such high sequence similarity across all ten strains exists, almost half of all parent ions in the molecular network are produced by strains from either Scotland or Antarctica, and not ubiquitously produced. This suggests that ecological differentiation has had a marked impact on the types of secondary metabolites that are produced by these taxonomically closely related strains.

Jensen *et al.* (2006) [67] investigated the biogeography of the genus *Salinispora* and concluded that due to the co-occurrence of three distinct clades, ecological differentiation is more important to speciation than biogeography. However, evidence of location specificity has been revealed when it was discovered that similar soil types contain related secondary metabolite genes [68]. It is important to note, however, that location may not be the only cause for the strain specific metabolomes in this study, due to the very extreme ecological differentiation between Antarctic and Scottish sediment. Strain specific metabolite production has been demonstrated recently by Duncan *et al.*, (2015) when 35 strains of three species of the marine actinomycete genus *Salinispora* were examined [45]. The data presented herein provides further evidence that strain specific metabolite production might be a wider occurrence across multiple bacterial taxa.

Rarefaction curves were computed using the MS/MS spectra, which were used to generate the molecular network (Figure S3). This reflects the diversity of the fragmentation spectra (and thus the parent ions within the dataset). The curve does not reach an asymptote, suggesting that significant metabolite diversity would be uncovered by investigating more strains. The molecular network by parent ion mass (Figure S4) indicates that 71.7*%* of the nodes represent small molecules, with masses between *m/z* 149 and 300. As expected the percentages decrease with increasing parent ion mass, with 14.4*%* of the networked ions between *m/z* 301 and 500, 9.8*%* between *m/z* 501 and 1000 and the largest parent ion masses (*m/z* 1001–2558) representing 3.9*%* of the networked ions. The molecular network exceeds 3500 individual nodes with mass ranges of *m/z* 149–2558. Previously, a network of 21 *Moorea*, *Lyngbya* and *Schizothrix* strains revealed 1948 nodes (ranging between *m/z* 300 and 2000) [44]. Additionally, a molecular network of 35 *Salinispora* strains resulted in 1137 parent ions; however, mass values less than *m/z* 300 were experimentally excluded [45]. The higher node counts of this study are likely a result of no exclusion of ions below *m/z* 300.

The molecular network (Figure S5) of the 38 strains by taxonomic identification (closest match within the NCBI BLASTx database, Table S1) revealed that the 21 *Bacillus* strains produced the highest number of parent ions (1349 ions, 39.7*%* of the network), followed by seven *Streptomyces* strains (477 ions, 14.1*%*), three *Kocuria* strains (215 ions, 6.3*%*) and two *Micromonospora* strains (156 ions, 4.6*%*). To take into account the varying number of strains of each genus, the average number of parent ions produced per strain was calculated for each genus and found to be 64.2, 68.1, 71.7 and 78.0 for *Bacillus*, *Streptomyces*, *Kocuria* and *Micromonospora* respectively. The four taxonomically unidentified bacteria (KP011, KP122, KP130 and KP133) that were grouped with the single *Micrococcus* isolate (KP018) resulted in 431 ions, making up 12.7*%* of the total parent ions of the molecular network. Only 395 parent ions (18.8*%*) crossed taxonomic boundaries and were produced by strains of more than two genera.

**Figure 6:**
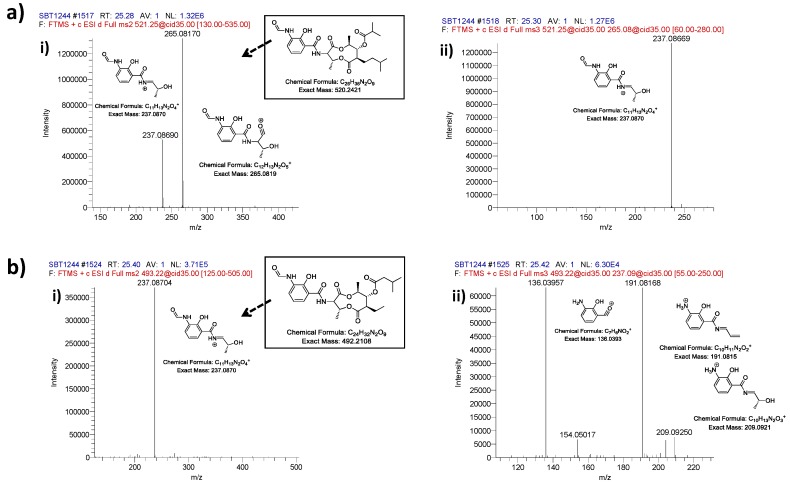
Fragmentation of parent ions that were tentatively assigned as antimycins showing fragmentation of (**a**) parent ion m/z *m/z* 521.24854 (Antimycin A3 or A7, C_26_H_36_N_2_O_9_) and (**b**) parent ion *m/z* 493.21734 (Antimycin A5, C_24_H_32_N_2_O_9_). MS*^2^* spectra (**i**) and MS*^3^* (**ii**) are annotated to illustrate the predicted fragmentation patterns of parent antimycins.

A heat map based on mass spectrometry profiles and molecular weight revealed three isolates with distinct patterns, KP130 (a taxonomically unidentified bacteria isolated from Maud Rise, Antarctica), KP044 (a *Streptomyces* strain isolated from St. Andrews sediment) and KP121 (a *Bacillus* strain from Bransfield Strait, Antarctica) (Figure S6). These three strains can be seen as principle component analysis (PCA) outliers (Figure S7), and were also clearly identified in the molecular network, demonstrating the complementary nature of metabolomic tools for secondary metabolite discovery, such as PCA, database searches (Marinlit and Antibase) and molecular networking (Figure S8, Table S2). The metabolites responsible for these unique heat map profiles were identified using PCA and found to be a series of polymers *m/z* 363–1911 with spacing of 86 Da (KP130); a series of the piscicides antimycins [69] known to be produced by *Streptomyces* spp. and which corresponded to ion *m/z* observed by Viegelmann *et al.*, (KP044) [70] and a series of unidentified small molecules below *m/z* 350 (KP121). The MS/MS fragmentation data for the antimycins from this study were compared with the data of Viegelmann *et al.* (Figure 6) [70]. The parent ion *m/z* 521.24854 showed fragmentation of the dilactone ring that generates a characteristic fragment ion at *m/z* 265.081 [M + H]^+^ and the parent ion *m/z* 493.21734 showed no characteristic fragment ion of *m/z* 265.081 as also found by Viegelmann *et al.*; however, an ion fragment of *m/z* 237.08704 was detected which may be the characteristic fragment ion minus a carbonyl group (Figure 6). However, many ions within the crude extracts do not match standards within the molecular networking database in addition to not having matches when comparing the MS data with the natural products databases Marinlit and Antibase during dereplication (data not shown). This allows ions to be prioritized for future chemical investigations and suggests that there is high potential for novel compound discovery from these fermentation extracts. In the future, combining whole genome sequencing of these strains will help reveal the biosynthetic gene clusters responsible for these molecular families using bioinformatic approaches such as peptidogenomics [47] and glycogenomics [46]. This comparison technique, has been used to discover several novel bioactive compounds including anticancer compound retimycin A [45], antifungal compound thanamycin [41] the vitroprocins produced by marine *Vibrio* sp. with antibiotic activity [71] and malyngamide C with a combination of antifungal and cytotoxic properties [49].

### 2.3. Bioactivity Testing

**Figure 7:**
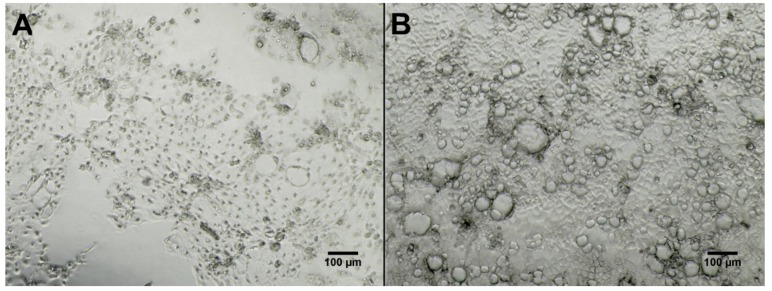
Micrographs (taken with EVOS XL Core Imaging system, 100× magnification in phase contrast) of Caco-2 cells seeded in 96 microplate wells after 48 h of exposure to metabolite extract of strain KP087 (**A**) at 10 µg/mL (viability test showed 37*%* viability for this well) and medium blank (**B**) at 10 µg/mL (viability test showed 100*%* viability for this well). Formation of apoptotic bodies can be seen in (**A**) as small black dots, as well as areas free of cells in the lower part of the picture as cells could no longer attach to plate. Cells exposed to medium blank (**B**) show full confluence where single cells cannot be distinguished.

A total of two blanks containing only ISP-2 media and organic extracts of all 38 strains selected for molecular networking were tested for cytotoxicity against two cell lines derived from different human tissue, *i.e.*, epithelial colon adenocarcinoma cells (Caco-2) and human fibroblasts originating from foreskin (HS27) with the additional testing of 29 extracts (and two media blanks) against human epithelial prostate cells (PNT2) (Table S3). There was no effect on the viability of “normal” human cell lines, PNT2 and HS27, at the concentrations tested (30 µg/mL and 100 µg/mL). While seven fermentation extracts were bioactive against colon adenocarcinoma cells at the concentration tested (50 µg/mL) (Table S3). These seven metabolite extracts were then further tested at several concentrations (10 µg/mL, 5 µg/mL, 1 µg/mL and 0.5 µg/mL). IC_50_ values were calculated that lowered cancer cell (Caco-2) viability to below 50*%* (Table 1). Five of the seven *Streptomyces* strains tested (KP022, KP044, KP082, KP087 and KP131) showed inhibition of cell viability values between 63%–75*%* at the highest concentration tested (50 µg/mL). Additionally, two *Bacillus* strains (KP039 and KP077) isolated from Scotland showed cell viability inhibition of 47*%* and 75*%* respectively when tested at the highest concentration (50 µg/mL). All metabolite extracts influenced cell viability in a concentration-dependent manner. Direct observation revealed morphological changes, *i.e.*, cell shrinkage and formation of apoptotic bodies (Figure 7). Both media blanks were tested and found to be inactive in the Caco-2 assay (Figure 7).

**Table 1:** IC_50_ values of seven metabolite extracts on Caco-2 cells. The values given reduce viability to 50*%* after 48 h of exposure.

Sample	IC_50_ (µg/mL)
KP022	6.2
KP039	55.5
KP044	0.1
KP077	1.6
KP082	30.0
KP087	6.1
KP131	31.4

Metabolites produced by *Streptomyces* and *Bacillus* strains have previously demonstrated anticancer bioactivities [46,72,73,74,75]. Our results suggest potential anticancer activity of the extracts (Table 1), as “normal” cell lines were not affected. While cultured immortalized cell lines are no substitute for *in vivo* toxicity studies, they can be used for screening studies as a control. Further analysis is needed to identify the causes for the cytotoxicity to cancer cells and to isolate compounds responsible for the observed bioactivity.

## 3. Experimental Section

### 3.1. Sample Collection and Processing

In total, four sediment samples were collected during research cruises occurring in March 2002 and February 2005 around the outer rim of the Weddell Sea, Antarctica. The sediment samples were collected by box-core from a depth range between 1166 m and 4652 m [76,77]. Sediment cores were collected from the Bransfield Strait (*62° 19.95 S 57° 54.00 W*), Wegener Canyon (*70° 31.49 S 14° 34.95 W*), the Maud Rise (*67° 30.99 S 0° 0.16 E*) and the Weddell Sea (*62° 57.68 S 27° 53.51 W*). These samples were stored in a temperature-controlled room (4 °C), located at the Scottish Association for Marine Science. Sediment samples for bacterial isolation were removed from the collected cores aseptically using a sterile spatula and sterile 50 mL centrifuge tube and processed immediately. Scottish samples were taken from an intertidal zone of: St. Andrews, on the East Coast of Scotland (in 2013); the Monach Isles in the Outer Hebrides (in 1990); and Calgary Bay on Mull on the West Coast of Scotland (in 1990).

### 3.2. Cultivable Bacterial Diversity

A combination of sediment pre-treatments, selective isolation media and plating techniques were used to target spore forming actinomycetes and *Bacillus* genera. A dry-stamp (DS) pre-treatment was employed [58], which involved initially drying the sediment samples in a Muffle furnace (60 °C; 1.5 h). The dried samples were subsequent stamped onto the surface of agar plates (containing selective media) using sterile foam tops (approx. 3 cm diameter), the stamping occurred around the media surface to create a serial dilution effect. A dilute-heat (DH) pre-treatment was also utilised, involving the dilution of 1 mL of wet sediment with 10 mL of sterile seawater, and then heating the sediment mixture (55 °C; 6:00 min) [58]. A 50–70 µL aliquot of the resulting suspension was inoculated onto the agar plates. Each pre-treatment was inoculated onto five isolation media: AI Agar [59], SC Agar [60], RH Agar [60], M4 Agar [61] and M3 Agar [60]. All media were prepared with 18 g·L^−1^ Instant Ocean^®^ (IO; Aquarium Systems, Sarrebourg, France) and sterilised by autoclaving. In total, five replicates of the DS pre-treatment and media combinations (AI, SC, RH, M4, M3) were plated for the Scottish isolates from all three sample locations, with five additional replicates for the DH pre-treatment and media combinations being plated from the St. Andrews location (resulting in 100 plates). Additionally, five replicates of DS pre-treatment and media combinations were plated for the Antarctic sediment samples (resulting in 125 plates). In total, 225 selective bacterial isolation plates were used. Plates were monitored over one month for the emergence of colonies exhibiting morphology typical of filamentous actinomycetes (includes a leathery substrate mycelia and the formation of aerial mycelia and spores). Colonies found to exhibit the desired morphologies were chosen for purification and initially subcultured onto fresh agar plates of the same media type from which they were isolated. Isolates were then phased onto International *Streptomyces* Project Medium 3 (ISP-3) [78]. Cultures were archived in 50*%* glycerol and stored (−80 °C).

### 3.3. Bacterial Fermentation and Metabolite Extraction

Cultures from agar plates were used to inoculate 7 mL of International *Streptomyces* Project Medium 2 (ISP-2) broth [78], supplemented with 18 g·L^−1^ IO, in culture tubes (150 × 15 mm). The seed cultures were grown over a one-week period (25 °C; shaking at 150 rpm). Fermentations (250 mL; ISP-2 medium/18 g·L^−1^ IO; 1000 mL Erlenmeyer flask), which included activated Diaion^®^ HP-20 resin, were inoculated with the seed culture and incubated under identical conditions for one week. The cells and resin were collected by centrifugation (5000× g, 10:00 min) in 50 mL Falcon tubes, where the supernatant was removed and the cells/resin combined in one Falcon tube, centrifuged and the supernatant removed. The cell/resin pellets were frozen (−80 °C; overnight), lyophilised and twice extracted (subsequently) with 20 mL of ethyl acetate (EtAOc) (Fisher Scientific, Leicester, UK) by agitation at 150 rpm for 1 h. Extracts were dried under nitrogen and the extract weights recorded.

### 3.4. Molecular Networking

High-resolution tandem mass spectrometry (HR-MS/MS) data for the 38 Antarctic and Scottish isolates was generated at the Strathclyde Institute of Pharmacy and Biomedical Science. Samples and ISP2 medium control samples were prepared at a concentration of 1 mg/mL in 80:20 MeOH: DCM as previously described [79]. Data dependent MS/MS experiments were carried out using a Finnigan LTQ Orbitrap coupled to a Surveyor Plus HPLC pump and autosampler (Thermo Fisher, Bremen, Germany) in positive ionization modes using a MS range of *m/z* 100–2000, a MS2 range of *m/z* 200–1500, a MSn range of *m/z* 0–1000 and 30,000 resolution as previously described [79]. LC-MS data was acquired using Xcalibur version 2.2.

HR-MS/MS raw data files were converted from .RAW to .mzXML file format using the Trans-Proteomic pipeline (Institute for Systems Biology, Seattle) [80], and clustered with MS-Cluster using Global Natural Products Social (GNPS) Molecular Networking [42,81,82,83]. A molecular network was created using the online workflow at GNPS. The data was filtered by removing all MS/MS peaks within ±17 Da of the precursor *m/z*. MS/MS spectra were window filtered by choosing only the top 6 peaks in the ±50 Da window throughout the spectrum. The data were then clustered with MS-Cluster with a parent mass tolerance of 2.0 Da and a MS/MS fragment ion tolerance of 0.5 Da to create consensus spectra. Further, consensus spectra that contained less than 1 spectra were discarded. A network was then created where edges were filtered to have a cosine score above 0.7 and more than 6 matched peaks. Further edges between two nodes were kept in the network if and only if each of the nodes appeared in each other’s respective top 10 most similar nodes. The spectra in the network were then searched against GNPS’s spectral libraries. The library spectra were filtered in the same manner as the input data. All matches kept between network spectra and library spectra were required to have a score above 0.7 and at least 6 matched peaks. To visualise the data, they were imported into Cytoscape suite (Version 3.0.2)[84] from this visualisation software, and nodes and edges were displayed (nodes correspond to a specific consensus spectrum; edges represent significant pairwise alignment between nodes). Cosine similarity scores, range from 0 to 1 (1 being identical spectra), were computationally combined as consensus spectra if more than six ion fragmentation spectra matched. A minimum cosine score of 0.7 was selected to subdue the clustering of different compound classes under the same molecular family when visualising the data as a network of nodes connected by edges [85]. Rarefaction curves portraying the diversity of MS/MS spectra were computed (with clusters affected equaling the number of clusters of which the file is a constituent) using GNPS [81].

### 3.5. Mass Spectrometry Chemical Dereplication

Mass spectrometry data was processed using a predefined metabolomics workflow described previously [79]. Raw positive ionisation mode data files were sliced using the MassConvert tool from ProteoWizard [86]. The sliced data sets were imported and processed in MZmine 2.10 [87] using settings described previously [79]. The processed data was incorporated into a macro embedded with the molecular formula data sets from Antibase^®^ (February 2013) and Marinlit^®^ (September 2013) which was used for peak identification and dereplication [76]. The data set was further analyzed using SIMCA-P V 13.0 using the unsupervised statistical analysis method, principal component analysis (PCA). Heat maps were generated using the programming software R (version ×64 2.15.2) (R Foundation for Statistical Computing, Vienna, Austria) using a script utilising the g-plot package to plot all *m/z* ratios from the sample set.

### 3.6. DNA Extraction, PCR Amplification, 16S rRNA Gene Sequencing of Bacterial Isolates

Pure bacterial isolates were cultured in ISP-2 media (5 mL) [78]. Genomic DNA was extracted from each cell pellet according to established protocols [88]. Polymerase chain reaction (PCR) amplification of the 16S rRNA gene was conducted in 50 μL volumes and consisted of the following: MyTaq^®^ Red 2× Master Mix (25 µL) (Bioline Reagents Ltd., UK), 0.2 μM 27f (5′-AGAGTTTGATCMTGGCTCA-3′) and 1492r (5′-TACGGYTACCTTGTTACGACT-3′) primers [89], and genomic DNA (1 μL of a 10^−1^ dilution). Thermocycling parameters consisted of initial denaturation at 95.0 °C for 5:00 min, 30 cycles of 94.0 °C for 0:10 min, 55.0 °C for 0:30 min and 72.0 °C for 3:00 min followed by a final extension at 72.0 °C for 10:00 min. PCR amplification was evaluated by agarose gel electrophoresis (0.5*%* agarose, 1 × TBE buffer strained with ethidium bromide) [90]. 16S rRNA gene amplicons were sequenced by LGC Genomics (Germany) using the primers 27f and 1492r. Sequences (≈1500 base pairs) were analysed and edited using the CodonCode Aligner software (Version 5.0.2) package (CodonCode Corporation, MA, USA).

### 3.7. Taxonomic Identification and Phylogeny of Bacterial Isolates

16S rRNA gene sequences for all 85 bacterial isolates were trimmed (0.05 error probability) using Geneious (version 8.1.5) [91]. Isolate 16S rRNA gene sequences were compared to sequences within the NCBI database using the Megablast Search Tool [92] within Geneious [91], and the results for closest match based on query coverage and sequence identity are shown in Table S1. Sequences were deposited in the NCBI database under the accession numbers KT200430-KT200510. Multiple alignment of all 85 16S rRNA gene sequences was achieved using a Clustal W [93] alignment with a IUB cost matrix (gap open cost 15, gap extend cost 6.66) using Geneious software (version 8.1.5) [91]. A consensus Neighbour-Joining tree [94] using a Jukes-Cantor [95] genetic distance model was computed using Geneious software (version 8.1.5) [91] with 1000 bootstrap re-samplings and a support threshold of 50*%*.

### 3.8. Bioactivity Testing of Fermentation Extracts Against Three Cell Lines

Caco-2 cells were purchased from ATCC and cultured in an incubator in 5*%* CO_2_ at 37 °C. Caco-2 cells were seeded at a density of 2 × 10^4^ cells/well in 100 µL of the growth medium Minimum Essential Medium alpha (MEMα) supplemented with 20*%* foetal bovine serum (FBS). After 48 h the growth medium was replaced with 80 µL of new medium that contained metabolite extracts at different concentrations (50 µg/mL, 10 µg/mL, 5 µg/mL, 1 µg/mL and 0.5 µg/mL). Cells were incubated for 48 hours before adding 20 µL of 50*%* PrestoBlue^®^ (resazurin based, Life Technologies) solution (diluted with growth media) for viability measurements. This mixture was incubated for 60 min before reading the fluorescence (570/585 nm (ex/em)). The percentage of treated cell viability was calculated and compared to untreated cells. The IC_50_ was calculated as the concentration of metabolites needed to reduce viability to 50*%*. Tests were done in triplicate. Additionally, cells were observed and micrographs taken using an EVOS XL Core imaging station (Life Technologies) with phase contrast before treatment, after 24 h and 48 h.

Normal fibroblasts derived from human foreskin (HS27 cells) and normal epithelial cells derived from human prostate (PNT2 cells) were obtained from ECACC (Sigma-Aldrich, Dorset, UK). HS27 cells were cultured in Dulbecco’s Modified Eagle’s Medium (DMEM) and PNT2 cells were cultured in RPMI 1640 media; both were supplemented with 10*%* (*v/v*) foetal bovine serum, 2 mM glutamine and 50 µg/mL penicillin/streptomycin solution (all Invitrogen, Paisley, UK) in a humidified incubator at 37 °C in the presence of 5*%* CO_2_. Cells were routinely passaged at 90%–95*%* confluence. Subsequently, cells were seeded at a concentration of 7500 (HS27) or 3750 (PNT2) cells/well in clear 96 flat-bottomed plates and allowed to adhere overnight. After that time, metabolite extracts were added at a final concentration of 30 µg/mL and allowed to incubate for 42 hours. Viability was determined using Alamar Blue^®^ (Thermo Fisher, Paisley, UK), according to the manufacturer’s instructions and incubated for a further 6 h. The resulting fluorescence was measured using a Wallac Victor 2 1420 multi-label counter (Perkin Elmer, Beaconsfield, UK), in fluorescence mode: excitation 560, emission 590. Vehicle treated control cells (media with 0.3*%* DMSO) were considered 100*%* viable against which metabolite extract treated cells (at a concentration of 30 µg/mL, at least *n* = 2) were compared. All results were confirmed microscopically.

## 4. Conclusions

This study is, to our knowledge, the first to use molecular networking to address the influence of bacterial isolation location on secondary metabolite production within the marine environment. The results accentuated the importance of bacterial isolation location when bioprospecting for novel bioactive compounds. In this study, it was found that 40*%* and 42*%* of parent ions were unique to Antarctic or Scotland, respectively (with only 8*%* shared between these locations), implying a high degree of biogeographic influence upon secondary metabolite production. Molecular networking combined with PCA metabolomics based dereplication were shown to be valuable tools for microbial ecologists and drug discovery chemists alike, where the matching of identical as well as related molecules allows metabolite patterns to be inferred and compounds to be prioritized for further chemical investigation. Comparative metabolomics aided in the metabolite dereplication of over 3500 parent ions from these understudied marine sources and has provided targets for further purification. Furthermore, due to the success of actinomycete bioactive metabolites for applications in biotechnology and biomedicine, the isolation of 13 *Streptomyces* strains (including two from Antarctic sediment) and the generation of metabolite extracts with activity against epithelial colon adenocarcinoma cells is highly desirable.

## Supplementary Materials

The following are available online at www.mdpi.com/2218-1989/6/1/2/s1, Figure S1: A total of sixty-seven bacteria by Scottish isolation location and media, Figure S2: A total of eighteen bacteria by Antarctica isolation location and media, Figure S3: Rarefaction curve of MS/MS spectra diversity for the molecular network of 3558 parent ions from 38 bacteria strains (Figure 2) where clusters affected is the number of spectra in each data file (strain) and dataset counts is the number of strains added, Figure S4: Molecular network by parent ion m/z, comprising 3558 nodes, colours indicate parent ion mass ranges from m/z 149 to 2558 as indicated in the LEGEND, Figure S5: Molecular Network comprising 3395 nodes, Figure S6: Heat map of 38 bacterial fermentation extracts based on mass spectrometry data indicating the distinct metabolic profiles amongst the strains. The strains observed as outliers in PCA are labelled with an asterisk *, Figure S7: (a) Principle component analysis scoring plot of 38 bacterial fermentation extracts based on mass spectrometry data indicating outlier strains with novel chemistry; (b) Loadings plot indicating metabolites present in outlying extracts responsible for separation observed in scoring plot, Figure S8: Molecular network using HR-MS/MS data of fermentation extracts from 38 bacteria strains,Table S1: Taxonomic identification of 85 bacterial isolates, derived through the NCBI (National Centre for Biotechnology Information) Megablast Search Tool (BLASTx) database, Table S2: Correlation of parent ions contributing to strains identified as outliers in PCA analysis (Figure S6) to parent ions in the molecular network (Figure 2) as depicted in Figure S7 boxes A–C, Table S3: Bioactivity testing of fermentation extracts from 38 strains and two media blank crude extracts against epithelial colon adenocarcinoma cells (Caco-2) and “normal” human fibroblasts (HS27) and prostate cells (PNT2a).
