# Supplementary material for: Using Molecular Networking for Microbial Secondary Metabolite Bioprospecting
Source: Metabolites. 2016 Jan 8;6(1):2. doi: 10.3390/metabo6010002 (PMC4812331; doi:10.3390/metabo6010002)
Supplement: Supplementary file 1 [file metabolites-06-00002-s001.pdf]

Supplementary Materials: Using Molecular Networking for Microbial Secondary Metabolite Bioprospecting

Kevin Purves, Lynsey Macintyre, Debra Brennan, Guðmundur Ó. Hreggviðsson, Eva Kuttner, Margrét E. Ásgeirsdóttir, Louise C. Young, David H. Green, Ruangelie Edrada-Ebel and Katherine R. Duncan

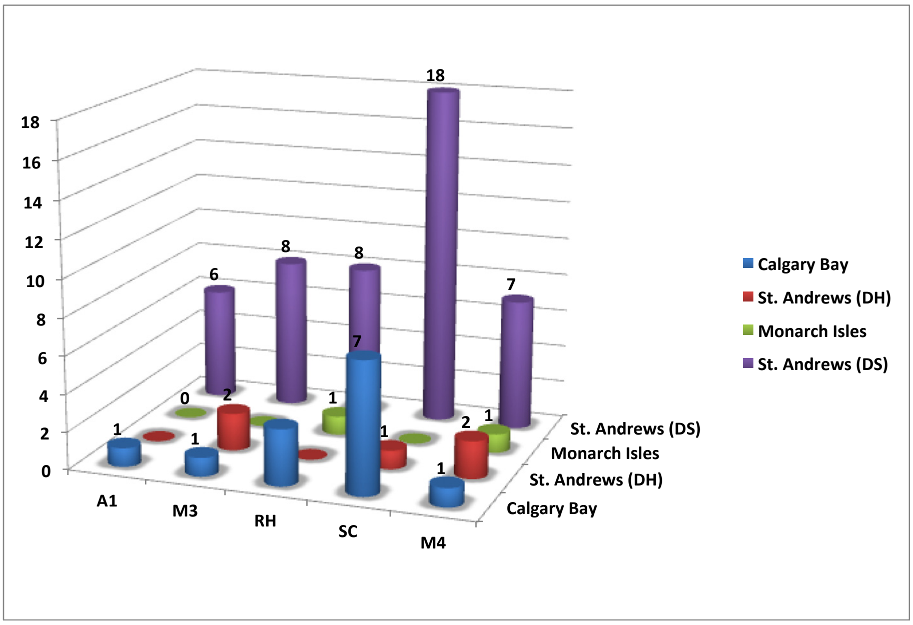

Figure S1. A total of sixty-seven bacteria by Scottish isolation location and media.

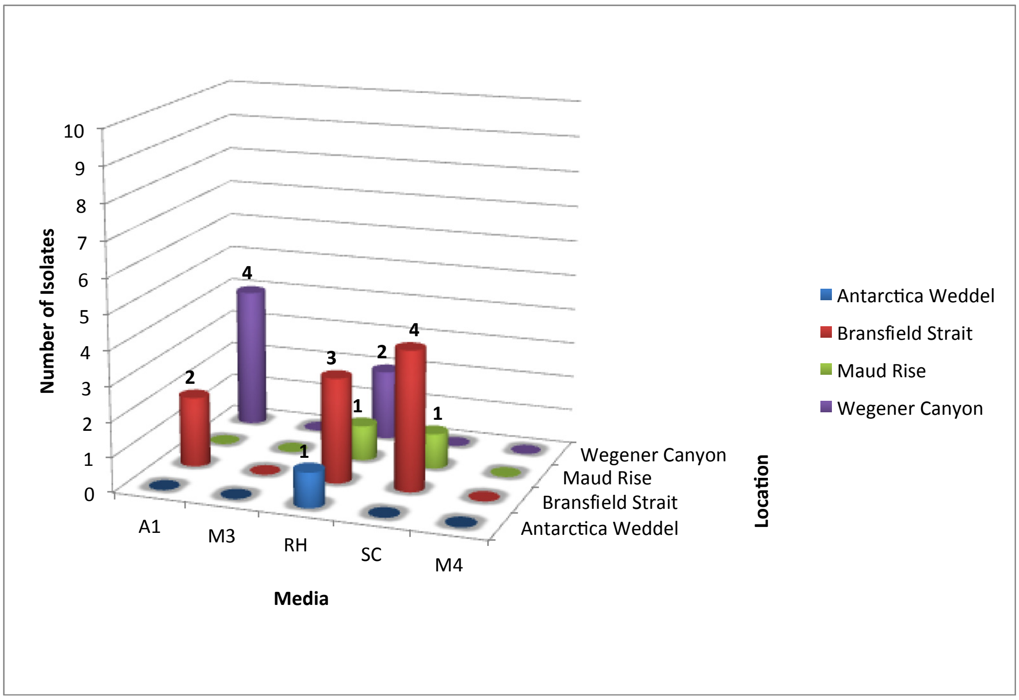

Figure S2. A total of eighteen bacteria by Antarctica isolation location and media.

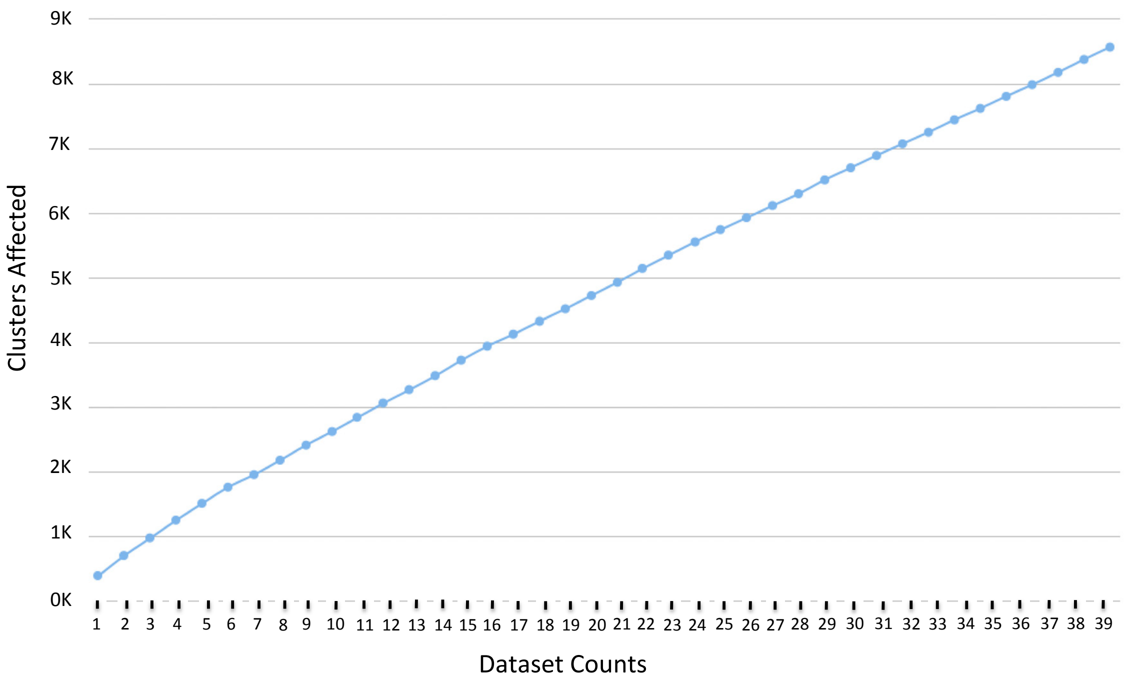

Figure S3. Rarefaction curve of MS/MS spectra diversity for the molecular network of 3558 parent ions from 38 bacteria strains (Figure 2) where clusters affected is the number of spectra in each data file (strain) and dataset counts is the number of strains added.

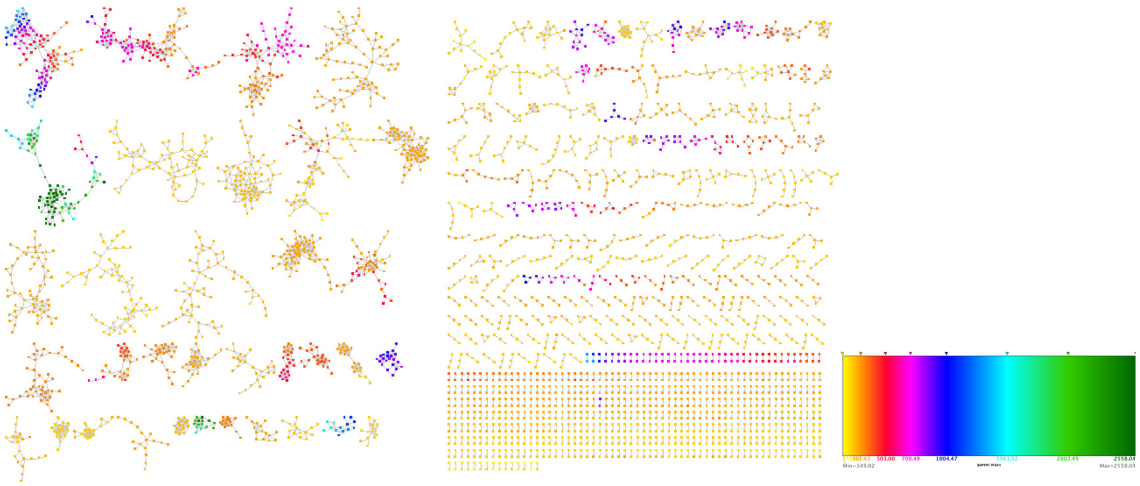

Figure S4. Molecular network by parent ion *m*/*z*, comprising 3558 nodes, colours indicate parent ion mass ranges from *m*/*z* 149 to 2558 as indicated in the LEGEND.

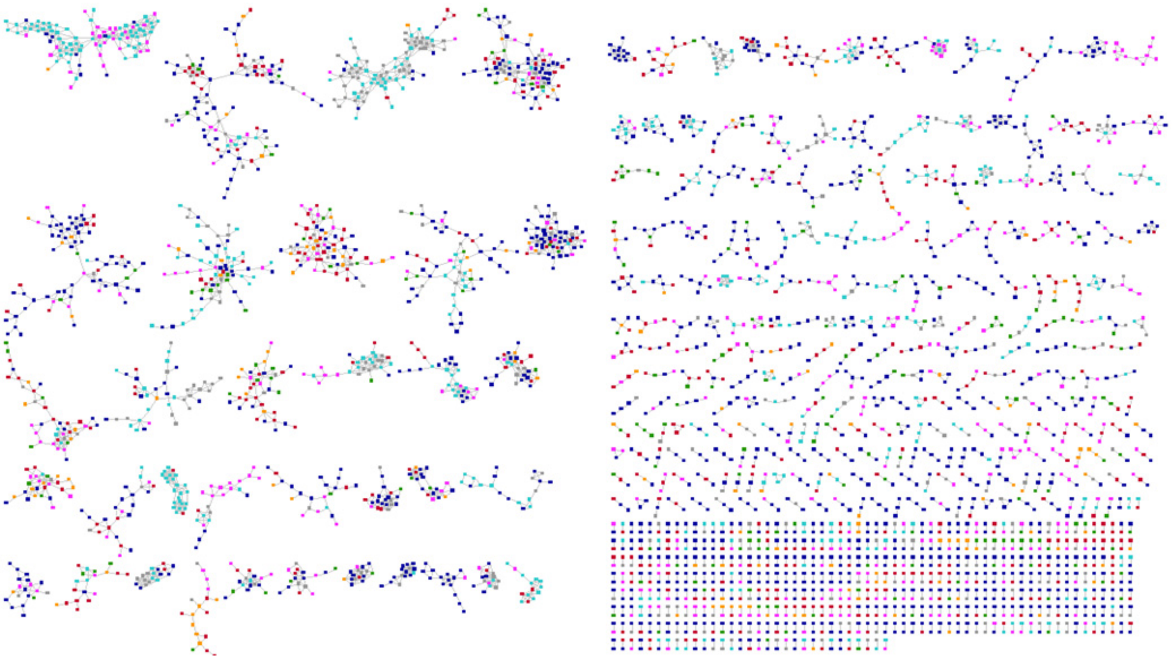

Figure S5. Molecular Network comprising 3395 nodes. The nodes are colour coded by strain taxonomy at the genus level: 21 *Bacillus* spp. (dark blue), seven *Streptomyces* spp. (red); three *Kocuria spp.* (green); two *Micromonospora* spp. (orange); four unidentified and one *Micrococcus* sp. (pink); compounds shared between taxa (light blue) and media components (grey).

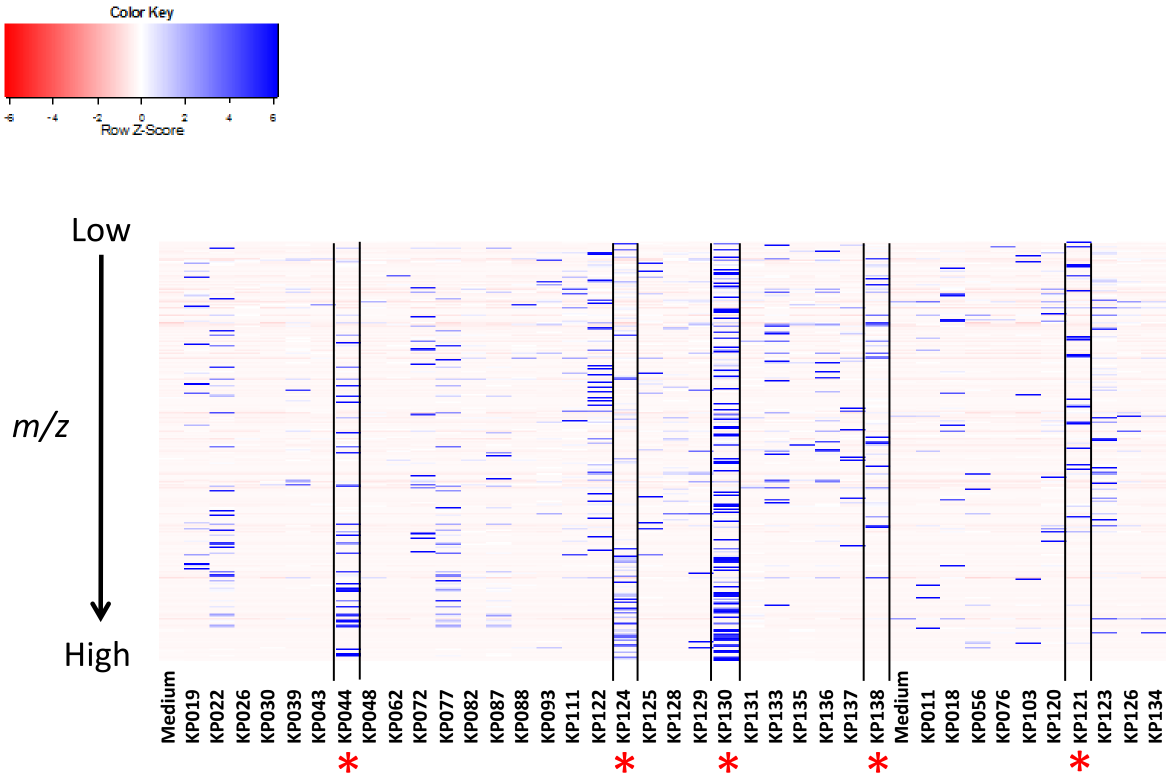

Figure S6. Heat map of 38 bacterial fermentation extracts based on mass spectrometry data indicating the distinct metabolic profiles amongst the strains. The strains observed as outliers in PCA are labelled with an asterisk *.

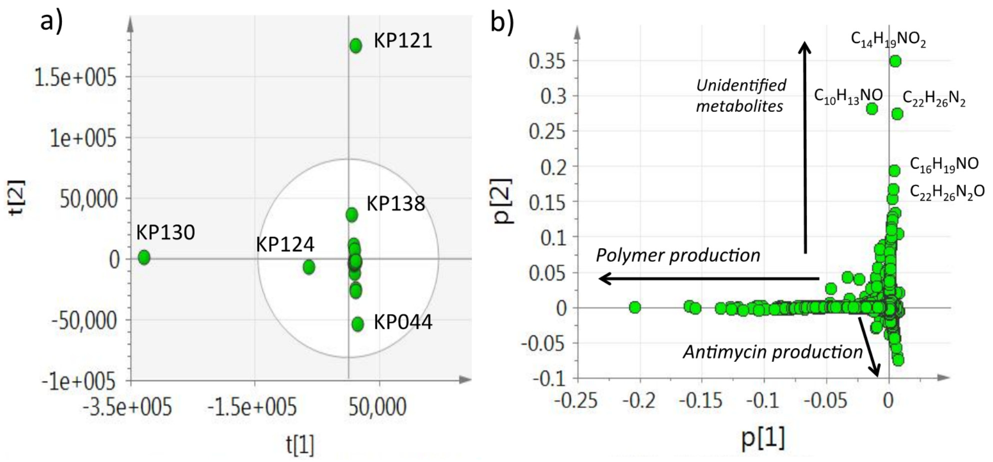

Figure S7. (**a**) Principle component analysis scoring plot of 38 bacterial fermentation extracts based on mass spectrometry data indicating outlier strains with novel chemistry; (**b**) Loadings plot indicating metabolites present in outlying extracts responsible for separation observed in scoring plot.

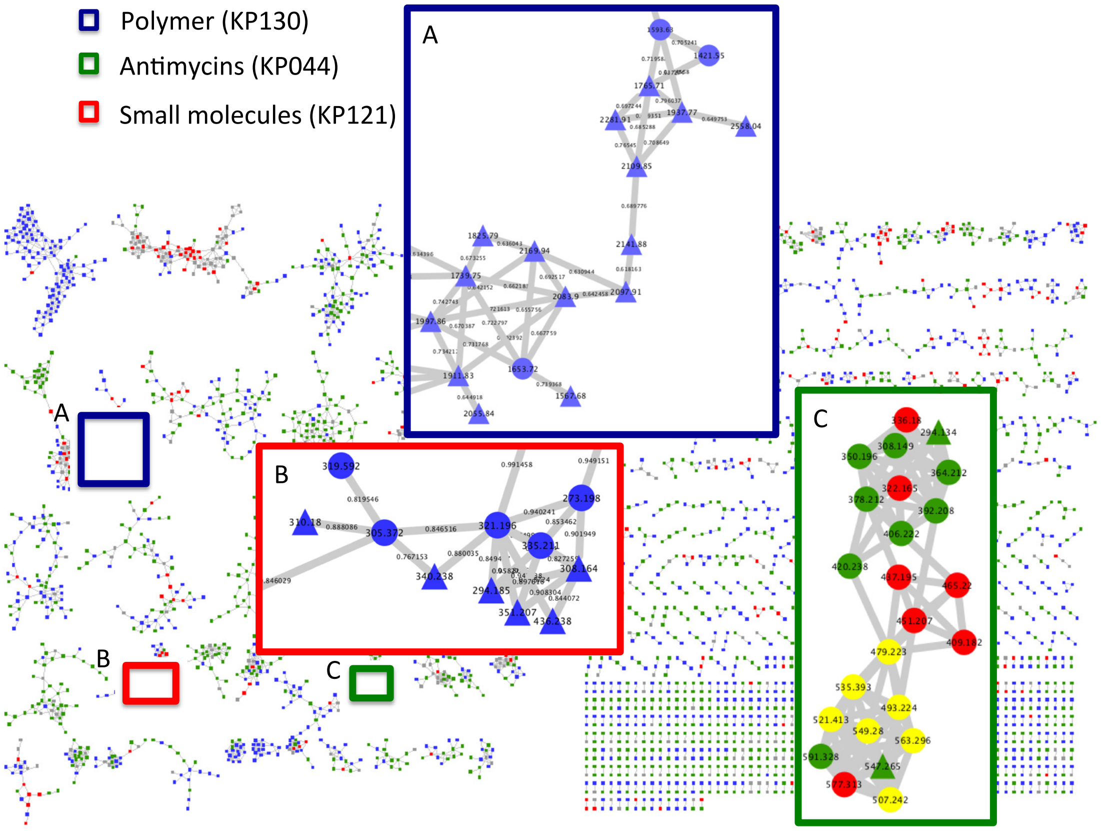

Figure S8. Molecular network using HR-MS/MS data of fermentation extracts from 38 bacteria strains (Figure 2), highlighting metabolites that have contributed to the strains being identified as outliers in PCA analysis (Figure S6). Metabolites include (**A**) a polymer series produced by strain KP130 (triangle nodes) and strains KP124 and KP130 (circle nodes) (**B**) small molecules below *m/z* 350) produced by strain KP121 (triangle nodes) and up to seven strains including KP121 (circle nodes) and (**C**) antimycins (yellow nodes) produced by strain KP044 and up to eight strains including KP044. All nodes depicted in boxes A–C are detailed in Table S2.

Table S1. Taxonomic identification of 85 bacterial isolates, derived through the NCBI (National Centre for Biotechnology Information) Megablast Search Tool (BLASTx) database. The query coverage (QC) is a measure of the coverage of the isolated 16S rRNA gene sequences against the query matches, whilst the sequence identity (ID) is the percentage identity to the database gene sequence. References marked with a * indicate isolates chosen for chemical investigation.

Reference	Accession Number	Sequence Length (bp)	BLASTn Hit	QC %	ID %	Accession Number of Closest BLASTn Hit	Location	Media	Pre-Treatment
KP011 *	-	-	-	-	-	-	St. Andrews	SC	DH
KP018 *	KT200430	1049	*Micrococcus yunnanensis*	99.81	100	NR_116578	Monach Isles	RH	DH
KP019 *	KT200431	1118	*Micromonospora saelicesensis*	100	99.6	NR_042312	Monach Isles	M4	DH
KP020	KT200432	1153	*Bacillus simplex*	98.88	98.6	NR_114919	St. Andrews	AI	DS
KP021	KT200433	1160	*Bacillus simplex*	99.15	98.7	NR_114919	St. Andrews	AI	DS
KP022r*	KT200434	1058	Streptomyces coelicolor	100	99.4	NR_116633	St. Andrews	AI	DS
KP023	KT200435	1134	*Bacillus* sp.	99.12	99.6	HM567027	St. Andrews	AI	DS
KP024	KT200436	1162	*Bacillus* sp.	100	99.8	KJ734005	St. Andrews	AI	DS
KP025	KT200437	1136	*Bacillus megaterium*	99.65	98.2	KJ526880	St. Andrews	AI	DS
KP026 *	KT200438	1209	*Bacillus aryabhattai*	99.92	99.7	LN827734	St. Andrews	M3	DS
KP027	KT200439	1137	*Streptomyces somaliensis*	99.74	99.9	KF973303	St. Andrews	RH	DS
KP028	KT200440	1135	*Bacillus licheniformis*	99.04	98.9	KJ842640	St. Andrews	RH	DS
KP030 *	KT200441	1220	*Bacillus licheniformis*	100	99.7	KC342874	St. Andrews	RH	DS
KP031	KT200442	1150	*Bacillus flexus*	99.05	98.9	NR_113800	St. Andrews	RH	DS
KP032	KT200443	1013	*Bacillus licheniformis*	97.87	98.7	KC813166	St. Andrews	RH	DS
KP036	KT200444	1175	*Paenibacillaceae* sp.	98.9	99.6	FM174172	St. Andrews	RH	DS
KP038	KT200445	1158	*Bacillus simplex*	98.72	98	HF584767	St. Andrews	RH	DS
KP039 *	KT200446	1137	*Bacillus pumilus*	100	99.7	KP224308	St. Andrews	RH	DS
KP040	KT200447	1129	*Bacillus licheniformis*	98.78	99.5	KC519411	St. Andrews	SC	DS
KP041	KT200448	1122	*Bacillus simplex*	99.29	99.9	NR_114919	St. Andrews	SC	DS
KP043 *	KT200449	1138	*Micromonospora saelicesensis*	99.74	99.8	JN862845	St. Andrews	SC	DS
KP044 *	KT200450	1124	*Streptomyces coelicolor*	100	99.6	NR_116633	St. Andrews	SC	DS
KP045	KT200451	1120	*Bacillus licheniformis*	99.29	100	KJ842635	St. Andrews	SC	DS
KP048 *	KT200452	1106	*Streptomyces albidoflavus*	100	99.6	LN626361	St. Andrews	SC	DS
KP050	KT200453	1153	*Bacillus licheniformis*	99.14	99.7	KF896091	St. Andrews	SC	DS
KP051	KT200454	961	*Bacillus licheniformis*	100	97.8	JN366714	St. Andrews	SC	DS
KP052	KT200455	1140	*Bacillus licheniformis*	99.04	99.7	KJ842635	St. Andrews	SC	DS
KP053	KT200456	1125	*Bacillus* sp.	98.94	100	KM675938	St. Andrews	SC	DS
KP055	KT200457	1123	*Bacillus* sp.	99.64	99.8	EU584553	St. Andrews	SC	DS
KP056 *	KT200458	1063	*Bacillus licheniformis*	99.53	99.8	KJ842640	St. Andrews	SC	DS

KP057	KT200459	1125	*Bacillus* sp.	99.03	99.8	KM675938	St. Andrews	SC	DS
KP058	KT200460	1131	*Bacillus* sp.	98.78	98.6	KM369837	St. Andrews	SC	DS
KP060	KT200461	1138	*Micromonospora sp.*	99.3	99	KF494813	St. Andrews	SC	DS
KP061	KT200462	1105	*Verrucosispora sp.*	96.17	98	JF346418	St. Andrews	SC	DS
KP062 *	KT200463	1071	*Bacillus licheniformis*	100	99.3	HG799978	St. Andrews	SC	DS
KP063	KT200464	1110	*Micromonospora* sp.	99.37	99.9	JQ782955	St. Andrews	SC	DS
KP064	KT200465	1105	*Streptomyces fungicidicus*	99.37	99.8	KF990583	St. Andrews	M4	DS
KP065	KT200466	1091	*Streptomyces albidoflavus*	100	99.3	LN626360	St. Andrews	M4	DS
KP066	KT200467	1151	*Bacillus marisflavi*	97.29	98.2	HE804783	St. Andrews	M4	DS
KP067	KT200468	1090	*Bacillus sp.*	99	100	KF933696	St. Andrews	M4	DS
KP069	KT200469	1092	*Streptomyces fungicidicus*	99.73	99.8	KF742498	St. Andrews	M4	DS
KP070	KT200470	1103	*Streptomyce violascens*	99.91	99.5	KJ571044	St. Andrews	M4	DS
KP071	KT200471	1101	*Bacillus* sp.	98.92	99.6	KC887502	Calgary Bay	AI	DS
KP072 *	KT200472	1157	*Bacillus simplex*	99.91	98.9	NR_114919	Calgary Bay	RH	DS
KP073	KT200473	1182	*Bacillus simplex*	99.33	99.7	KF818647	Calgary Bay	RH	DS
KP074	KT200474	1230	*Bacillus licheniformis*	99.19	99.3	KF896091	Calgary Bay	SC	DS
KP076 *	KT200475	1135	*Bacillus hwajinpoensis*	100	99.4	NR_025264	Calgary Bay	SC	DS
KP077 *	KT200476	1096	*Bacillus hwajinpoensis*	100	100	NR_025264	Calgary Bay	SC	DS
KP079	KT200477	1099	*Bacillus hwajinpoensis*	99.01	99.9	KF933706	Calgary Bay	SC	DS
KP081	KT200478	1229	*Bacillus licheniformis*	99.19	99.5	EU816690	Calgary Bay	SC	DS
KP082 *	KT200479	1059	*Streptomyces daghestanicus*	100	99.6	KM036200	Calgary Bay	SC	DS
KP083	KT200480	1216	*Bacillus hwajinpoensis*	99.26	99.1	KF933706	Calgary Bay	SC	DS
KP086	KT200481	1097	*Bacillus hwajinpoensis*	99.1	99.9	KM369837	Calgary Bay	M4	DS
KP087 *	KT200482	1047	*Streptomyces albidoflavus*	100	99.8	LN626361	St. Andrews	M3	DH
KP088 *	KT200483	1105	*Bacillus hwajinpoensis*	99.82	99.6	KF933706	St. Andrews	M3	DS
KP091	KT200484	1068	*Bacillus licheniformis*	99.53	99.9	KC456634	St. Andrews	M3	DS
KP092	KT200485	1194	*Bacillus altitudinis*	99.58	99.9	NR_118439	St. Andrews	M3	DS
KP093 *	KT200486	1137	*Bacillus licheniformis*	99.82	99.5	KF896091	St. Andrews	M3	DS
KP094	KT200487	1075	*Verrucosispora* sp.	100	99.9	KM370030	St. Andrews	M3	DS
KP095	KT200488	1245	*Streptomyces exfoliatus*	100	99	NR_041229	St. Andrews	M3	DH
KP097	KT200489	1075	*Bacillus* sp.	99.17	100	KM67938	St. Andrews	M3	DS
KP098	KT200490	1099	*Paenibacillus xylanexedens*	99.37	99	KF562253	Calgary Bay	RH	DS
KP101	KT20049t	1073	*Staphylococcus warneri*	99.17	100	HG799952	St. Andrews	M3	DS
KP103(2) *	KT200492	1252	*Bacillus hwajinpoensis*	99.04	98.7	NR_025265	Calgary Bay	M3	DS
KP105	KT200493	399	*Paenibacillus pasadenensis*	95.89	96.2	NR_113987	St. Andrews	M4	DS
KP107	KT200494	1081	*Paenibacillaceae sp.*	99.17	99.8	FM174172	St. Andrews	M4	DH

KP109	KT200495	1258	*Bacillus subtilis*	99.05	98.6	JF701929	St. Andrews	M4	DH
KP111 *	KT200496	990	*Kocuria* sp.	99.9	99.9	KM507644	Antarctica Weddel	RH	DS
KP120 *	KT200497	1133	*Bacillus licheniformis*	100	99.8	NR_118996	Bransfield Strait	RH	DS
KP121 *	KT200498	1095	*Bacillus licheniformis*	100	99.5	NR_118996	Bransfield Strait	RH	DS
KP122 *	-	-	-	-	-	-	Bransfield Strait	SC	DS
KP123 *	KT200499	1136	*Bacillus* sp.	100	99.6	KF896091	Bransfield Strait	SC	DS
KP124 *	KT200500	1026	*Bacillus sonorensis*	99.9	99.6	KP164479	Bransfield Strait	RH	DS
KP125 *	KT200501	797	*Bacillus* sp.	100	99.7	KM675938	Bransfield Strait	AI	DS
KP126 *	KT200502	1137	*Bacillus* sp.	99.91	99.5	KF896091	Bransfield Strait	AI	DS
KP128 *	KT200503	1119	*Bacillus licheniformis*	100	99.6	HG799978	Bransfield Strait	SC	DS
KP129 *	KT200504	1120	*Bacillus* sp.	100	99.8	KF896091	Bransfield Strait	SC	DS
KP130 *	-	-	-	-	-	-	Maud Rise	RH	N/A
KP131 *	KT200505	1162	*Streptomyces albidoflavus*	99.91	99.3	LN626361	Maud Rise	SC	N/A
KP134 *	KT200506	1125	*Streptomyces champavatii*	100	99.8	NR_115669	Wegener Canyon	AI	DS
KP133 *	-	-	-	-	-	-	Wegener Canyon	AI	DS
KP135 *	KT200507	1023	*Kocuria rhizophila*	100	99.7	NR_026452	Wegener Canyon	RH	DS
KP136 *	KT200508	1143	*Kocuria rhizophila*	100	99.7	EU554435	Wegener Canyon	RH	DS
KP137 *	KT200509	1093	*Bacillus licheniformis*	99.73	99.7	JX625995	Wegener Canyon	AI	DS
KP138 *	KT200510	1120	*Bacillus* sp.	100	99.7	KF896091	Wegener Canyon	AI	DS

Table S2. Correlation of parent ions contributing to strains identified as outliers in PCA analysis (Figure S6) to parent ions in the molecular network (Figure 2) as depicted in Figure S7 boxes A–C.

Compound	Network *m/z*	PCA *m/z*	Network Strain
Polymer	1421.55		KP124, KP130
	1567.68		KP130
	1593.63		KP124, KP130
	1653.72		KP124, KP130
	1739.75		KP130
	1765.71		KP130
	1825.79		KP130
	1911.83		KP130
	1937.77		KP130
	1997.86		KP130
	2055.84		KP130
	2083.9		KP130
	2097.91		KP130
	2109.85		KP130
	2141.88		KP130
	2169.94		KP130
	2281.91		KP130
	2558.04		KP130
Unknown small molecules <350 m/z	273.198		KP121, KP122
	294.185		KP121
	305.372		KP121, KP122, KP123, KP126, KP128, KP130, KP136
	308.164		KP121
	310.18		KP121
	319.592	319.217	KP121, KP122, KP126, KP138
	321.196	321.196	KP121, KP122
	335.211	335.212	KP121, KP136, KP130, KP138
	340.238		KP121
	351.207		KP121
	436.238		KP121
Antimycin A6, A18	479.223	479.203	KP077, KP087, KP044
Antimycin A5	493.224	493.218	KP022, KP077, KP044
Antimycin A4b, A20	507.242	507.234	KP022, KP077, KP087, KP044
Antimycin A3b, A4a, A7a, A7b	521.413	521.249	KP131, KP138, KP022, KP077, KP087, KP044
Antimycin A2a, A2b, A8a, A8b, A11, A17	535.393	535.265	KP124, KP131, KP022, KP077, KP087, KP044
Antimycin A0b, A1b, A12, A13, A19	549.28	549.280	KP131, KP138, KP022, KP077, KP087, KP039, KP044, KP082
Antimycin A0a, A0c, A0d, A10a, A10b, A14, A15	563.296	563.296	KP022, KP077, KP087, KP044

Table S3. Bioactivity testing of fermentation extracts from 38 strains and two media blank crude extracts against epithelial colon adenocarcinoma cells (Caco-2) and "normal" human fibroblasts (HS27) and prostate cells (PNT2a). Activity is measured in percentage of viable cells. nt = not tested, red font indicates results considered active.

Cell Line	CaCo-2 Viability (Percentage)	HS27 Viability (Percentage)		PNT2a Viability (Percentage)	
Extract Concentration µg/mL	50	30	100	30	100
Extract Reference					
KP111	101	86	87	91	74

KP122	106	98	99	96	92
KP125	91	90	91	100	88
KP128	93	97	97	98	95
KP129	97	100	100	97	97
KP022	25	106	113	91	87
KP026	93	97	95	94	94
KP043	89	96	94	96	94
KP048	107	98	97	96	93
KP077	23	104	109	74	66
KP087	24	107	104	92	89
KP093	108	101	98	96	97
KP019	107	98	90	91	83
KP030	110	98	101	94	94
KP039	37	112	104	94	93
KP044	22	114	110	91	86
KP072	105	107	104	81	89
KP082	20	107	101	93	94
KP088	53	102	99	92	95
KP124	100	100	93	88	92
KP062	97	101	101	94	98
KP131	22	106	92	91	92
KP138	72	102	102	90	96
KP133	110	98	98	94	96
KP137	112	99	95	90	92
KP136	109	93	95	93	95
KP135	107	95	98	93	93
KP130	103	103	97	95	98
Media Blank	86	95	97	95	94
KP134	104	97	99	nt	nt
KP123	102	97	100	nt	nt
KP018	105	82	90	nt	nt
KP076	100	94	101	nt	nt
KP011	105	98	100	nt	nt
KP056	104	97	101	nt	nt
KP103	100	94	98	nt	nt
KP120	93	82	88	nt	nt
KP121	101	109	108	nt	nt
KP126	102	97	101	nt	nt
Media Blank	107	103	103	nt	nt
